# Prostatic abscess with infected aneurysms and spondylodiscitis after transrectal ultrasound-guided prostate biopsy: a case report and literature review

**DOI:** 10.1186/s12894-021-00780-0

**Published:** 2021-01-21

**Authors:** Shunichiro Nomura, Yuka Toyama, Jun Akatsuka, Yuki Endo, Ryoji Kimata, Yasutomo Suzuki, Tsutomu Hamasaki, Go Kimura, Yukihiro Kondo

**Affiliations:** Department of Urology, Nippon Medical School, 1-1-5 Sendagi, Bunkyo-ku, Tokyo, 113-8603 Japan

**Keywords:** Spondylodiscitis, Aneurysm, Prostate biopsy

## Abstract

**Background:**

Transrectal ultrasonography (TRUS)-guided prostate biopsy is the conventional method of diagnosing prostate cancer. TRUS-guided prostate biopsy can occasionally be associated with severe complications. Here, we report the first case of a prostate abscess with aneurysms and spondylodiscitis as a complication of TRUS-guided prostate biopsy, and we review the relevant literature.

**Case presentation:**

A 78-year-old man presented with back pain, sepsis, and prostate abscesses. Twenty days after TRUS-guided prostate biopsy, he was found to have a 20-mm diameter abdominal aortic aneurysm that expanded to 28.2 mm in the space of a week, despite antibiotic therapy. Therefore, he underwent transurethral resection of the prostate to control prostatic abscesses. Although his aneurysm decreased to 23 mm in size after surgery, he continued to experience back pain. He was diagnosed as having pyogenic spondylitis and this was managed using a lumbar corset. Sixty-four days after the prostate biopsy, the aneurysm had re-expanded to 30 mm; therefore, we performed endovascular aneurysm repair (EVAR) using a microcore stent graft 82 days after the biopsy. Four days after the EVAR, the patient developed acute cholecystitis, and he underwent endoscopic retrograde biliary drainage. One hundred and sixty days after the prostate biopsy, all the complications had improved, and he was discharged. A literature review identified a further six cases of spondylodiscitis that had occurred after transrectal ultrasound-guided prostate biopsy.

**Conclusions:**

We have reported the first case of a complication of TRUS-guided prostate biopsy that involved prostatic abscesses, aneurysms, and spondylodiscitis. Although such complications are uncommon, clinicians should be aware of the potential for such severe complications of this procedure to develop.

## Background

Transrectal ultrasonography (TRUS)-guided prostate biopsy is the standard method of diagnosing prostate cancer [1]. TRUS-guided prostate biopsy is a relatively safe method that is usually well-tolerated by patients, although minor complications (such as pain, hematuria, hematospermia, and rectal hemorrhage) or, rarely, significant complications (such as sepsis, macroscopic hematoma, and urinary retention) can develop [2]. Here, we report the first case of prostatic abscess with aneurysms and spondylodiscitis after TURS-guided prostate biopsy, and contextualize this with a review of the literature.

## Case presentation

**Fig. 1:**
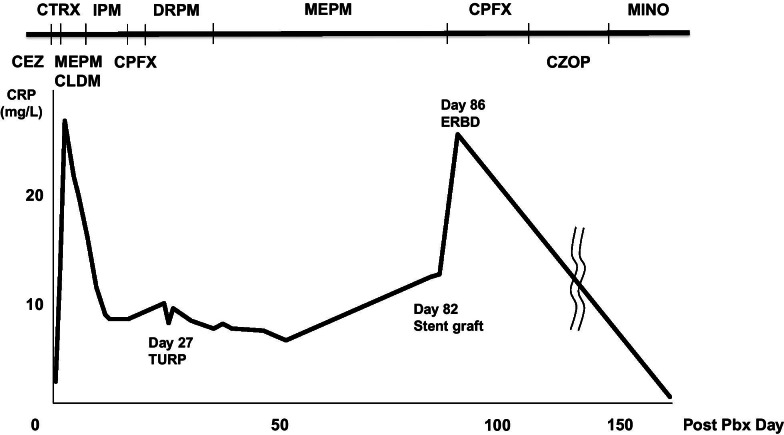
Clinical course and changes in serum C-reactive protein concentration. CEZ: cefazolin, CTRX: ceftriaxone, MEPM: meropenem, CLDM: clindamycin, IPM: imipenem, CPFX: ciprofloxacin, DRPM: doripenem, CZOP: cefozopran, MINO: minocycline

A 78-year-old man was hospitalized to undergo TRUS-guided prostatic biopsy because he had a serum prostate-specific antigen (PSA) concentration of 15.86 ng/ml. The TRUS-measured prostate volume was 41 ml. His medical history included coronary artery disease with previous intracoronary stenting. He received intravenous (IV) cefazolin 2 g daily as antimicrobial prophylaxis prior to the biopsy. The day after the biopsy, he had a fever (temperature 39.5 °C), but his vital signs were otherwise normal. Blood analyses revealed a white blood cell (WBC) count of 7100/μl. After 3 days, he was started on intravenous ceftriaxone and gamma-globulin 5 g because of a continuous fever and a high serum C-reactive protein (CRP) concentration (Fig. 1). After 5 days, a gram-negative bacillus was grown on blood culture. A digital rectal examination revealed an enlarged prostate. Computed tomography (CT) of the pelvis showed mild enlargement of the prostate. Therefore, the patient’s antibiotics were changed to meropenem 1.5 g and clindamycin to treat the sepsis of prostatitis, and a transabdominal catheter was placed into his urinary bladder. However, his condition worsened 6 days later and he had difficulty breathing, with an oxygen saturation of 85%, according to pulse oximetry, and a low pO2 (32.4 mmHg), according to arterial blood gas measurement. A chest X-ray showed air-space consolidation. Therefore, he was transferred to the intensive care unit for the diagnosis of acute respiratory distress syndrome (ARDS).

**Fig. 2:**
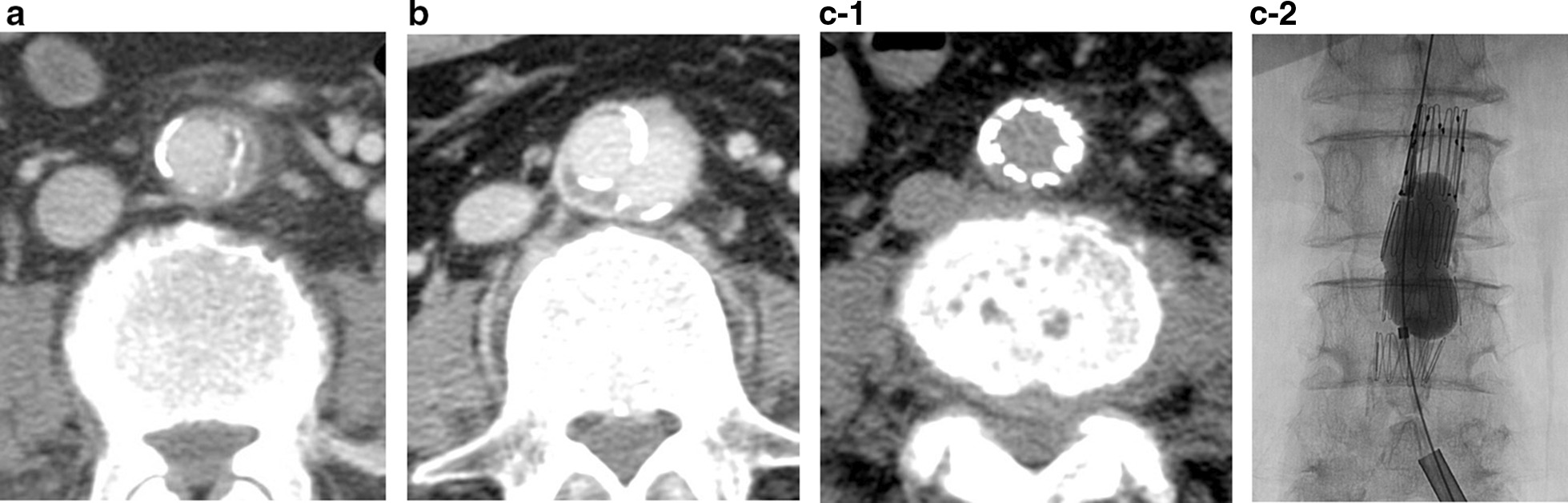
Images of the aortic aneurysm. **a** Initial computed tomography (CT) image of the abdomen/pelvis, demonstrating a 20-mm abdominal aortic aneurysm, with evidence of inflammatory changes, 20 days after prostate biopsy. **b** Repeat CT image revealing that the abdominal aortic aneurysm diameter has increased to 30 mm, 64 days after the prostate biopsy. **c** CT and angiography showing the successful exclusion of the aortic rupture by endovascular stent grafting

At this time, a further blood culture showed the presence of extended-spectrum-lactamase (ESBL)-producing *Escherichia coli* (*E. coli*), which was sensitive to imipenem; therefore, his antibiotics were changed accordingly. Twelve days later, rectal examination revealed a tender, fluctuant prostate consistent with a prostatic abscess. The CT images were consistent with the presence of three prostatic abscesses (< 1.5 cm each). Thirteen days after the prostate biopsy, his bacteremia had improved and his ARDS had resolved; therefore, he was transferred to the general ward. Twenty days after the prostate biopsy, an abdominal/pelvic CT scan was performed to identify any remaining prostatic abscesses. However, instead, the scan revealed the presence of an abdominal aortic aneurysm with surrounding periaortic inflammatory changes (Fig. 2a). Therefore, IV doripenem was administered. CT examination after 26 days showed no change in the prostatic abscesses, but the aneurysm had expanded to a diameter of 28.2 mm.

**Fig. 3:**
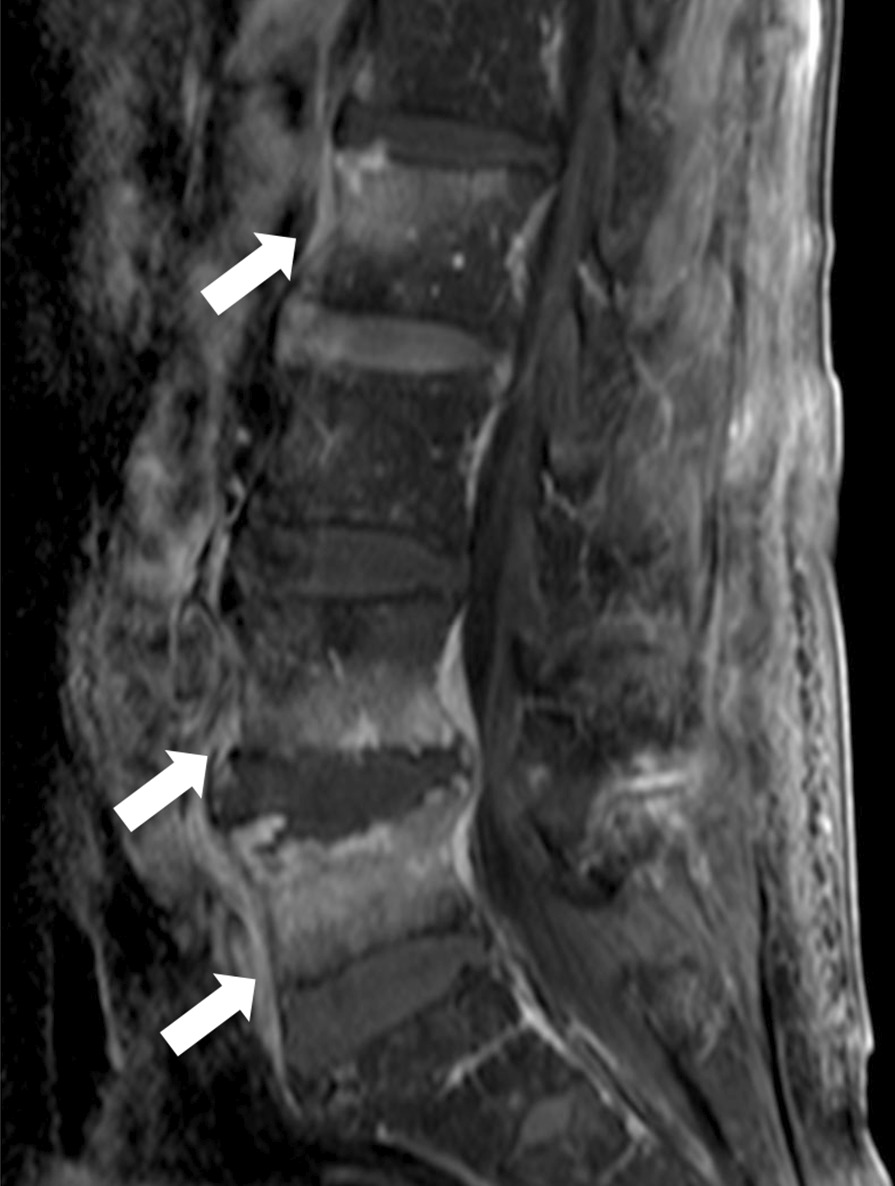
Lumbar images obtained 40 days after prostate biopsy. Contrast-enhanced T1-weighted magnetic resonance image of the lumbar spine shows contrast-enhancement of the L2, L4, and L5 vertebral bodies

Transurethral resection of the prostate was planned as a means of controlling prostatic abscesses for day 27 after the prostate biopsy. During the surgery, a whitish purulent substance discharged from the prostate. After surgery, the patient made excellent progress. Postoperative CT (31 days after prostate biopsy) showed that the aneurysm had a satisfactory appearance (23 mm diameter). However, he had also been experiencing back pain for 4 weeks, from day 11 after the prostate biopsy. Contrast-enhanced magnetic resonance imaging of the lumbar spine revealed contrast enhancement of the L2, L4, and L5 vertebral bodies (Fig. 3). Therefore, he was diagnosed as having pyogenic spondylitis and this was managed using a lumbar corset. Sixty-four days after the original biopsy, the aneurysm had re-expanded to 30 mm (Fig. 2b), but as the patient’s condition was unsatisfactory, surgery was not attempted. Instead, he underwent endovascular aneurysm repair (EVAR) using a microcore stent graft at our hospital 82 days after the prostate biopsy (Fig. 2c). Four days after the endovascular treatment, he developed a stomach ache and CT revealed acute cholecystitis. Therefore, endoscopic retrograde biliary drainage (ERBD) was performed and the patient’s antibiotic was changed to ciprofloxacin. By day 160 after the prostate biopsy, all the complications had improved, and the patient was discharged. He then underwent active surveillance for prostate cancer 1 year after the original diagnosis.

## Discussion and conclusions

To the best of our knowledge, prostate abscess with infected aneurysms and spondylodiscitis has not been reported in the literature as a complication of transrectal prostate biopsy. Furthermore, infected aneurysm alone is an extremely rare complication of TURS-guided prostate biopsy: only two cases have been reported previously [3].

Infected aneurysms can arise because of the hematogenous spread of bacteria, which cause aneurysmal changes in the arterial wall, and a pathogen can be further disseminated by this route to cause a prostate abscess. The gold-standard treatment of the disease remains surgical resection and debridement of the infected aorta, involving the placement of an interposition graft or extra-anatomic bypass, and long-term antibiotic therapy. However, this procedure is associated with high mortality of 13.3–40% [4, 5]. Therefore, EVAR has been developed as an elective open method of repair of an infected aneurysm, especially for high-risk surgical patients. Because the present patient was high-risk, we chose to treat the aneurysm using EVAR, and although he developed acute cholecystitis 4 days after the EVAR, his condition was improved by ERBD.

Spondylodiscitis is also an extremely rare complication of TURS-guided prostate biopsy. The incidence of spondylodiscitis ranges from 0.4 to 2.4/100,000 in Europe [6]. Six cases of spondylodiscitis following TRUS-guided prostate biopsy have been previously described in the English-speaking literature, (Table 1) and in all these cases the clinical symptoms were fever and backache. *E. coli* was the dominant cause of the spondylodiscitis, being identified in five of the cases, three of which were antibiotic-resistant. *Enterococcus faecalis* was identified as the causative agent in the remaining case. Here, we report the first case of spondylodiscitis that was caused by ESBL *E. coli*. Four of the seven reported cases had positive blood culture results. Lumbar vertebrae are most frequently affected in spondylodiscitis, as in five of the reported cases, including the present case. Risk factors to infection include immunosuppression, diabetes mellitus, and benign prostatic hypertrophy. Although three of the reported cases had risk factors, our patient had none of the risk factors. Five of the cases were treated using a surgical intervention, but the spondylodiscitis in the present case was treated by antibiotic therapy and spinal immobilization. Four of the cases also had an epidural abscess. However, all the identified lesions resolved, suggesting that such lesions have good prognoses.

**Table 1:** Review of the reported cases of spondylodiscitis after transrectal ultrasound-guided prostate biopsy

References	Age	Symptoms	Antimicrobial prophylaxis	Location	Susceptibility	Bacteremia
Taşdemiroğlu et al. [12]	53	Fever, backache	None	L2–3	*E. coli*	No
Karapolat et al. [13]	70	Fever, backache	Ciprofloxacin, Gentamicin	T6–7	*E. coli*	No
Kaya et al. [14]	59	Fever, backache	Ciprofloxacin	L4–5	*E. coli*	No
Dobson et al. [15]	69	Fever, backache	Ciprofloxacin	L2–3	Fluoroquinolone resistant *E. coli*	Yes
Hiyama et al. [16]	59	Fever, backache	Cefotiam	C7/T1	*Enterococcus faecalis*	Yes
Li et al. [17]	71	Fever, backache	Levofloxacin	L3–5	Multiple drug resisitant *E. coli*	Yes
Present case	78	Fever, backache	Cefazolin	L2, 4, 5	ESBL *E. coli*	Yes

Reference	Risk factor	Surgery	Complications		Outcome	
Taşdemiroğlu et al. [12]	DM	Hemilaminectomy	Epidural abscess		Resolution	
Karapola et al. [13]	BPH	Bone graft reconstruction	None		Resolution	
Kaya et al. [14]	None	Hemilaminectomy	Epidural and intradiscal abscess		Resolution	
Dobson et al. [15]	None	Aspiration	Epidural abscess		Resolution	
Hiyama et al. [16]	None	Aortic valve displacement	Endocarditis		Resolution	
Li et al. [17]	BPH	Laminectomies	Epidural and psoas muscle abscess		Resolution	
Present case	None	TURP, EVAR	Prostate abscesses, infected aneurysm		Resolution	

*E. coli Escherichia coli*, *ESBL* extended-spectrum lactamase, *DM* diabetes mellitus, *BPH* benign prostatic hypertrophy, *TURP* transurethral resection of the prostate, *EVAR* endovascular aneurysm repair

Fluoroquinolones, and particularly ciprofloxacin, are widely used prophylactically alongside TRUS-guided prostate biopsy. However, the incidence of infectious complications associated with prostate biopsy has significantly increased in recent years [7]. Recent studies have shown that approximately half of post-biopsy infections are resistant to fluoroquinolone, and many are also resistant to other antibiotics [8]. Therefore, to prevent post-biopsy infection, we should consider selectively targeting antibiotic prophylaxis by performing a pre-biopsy rectal culture. Taylor et al. reported that targeted antimicrobial prophylaxis is associated with a notable reduction, in the incidence of infectious complications associated with TRUS-guided prostate biopsy that were caused by fluoroquinolone-resistant organisms, as well as a reduction in the overall cost of care. No infectious complications arose in the 112 men who received targeted antimicrobial prophylaxis, whereas there were nine cases (including one of sepsis) among the 345 men who were on empirical therapy [9]. Other proposed procedures include transperineal prostate biopsy and selectively augmented prophylaxis with two antibiotics in higher risk patients [10, 11].

In conclusion, we have reported the first case of prostatic abscess with aneurysm and spondylodiscitis after TRUS-guided prostate biopsy. Although this combination of complications is extremely rare, we should be aware of the possibility that severe complications can arise following this procedure.

## Data Availability

Records and data pertaining to this case are held in the patient’s secure medical records in Nippon Medical School Hospital.

## Abbreviations

TRUS: Transrectal ultrasonography
IV: Intravenous
CRP: C-reactive protein
CT: Computed tomography
EVAR: Endovascular aneurysm repair
